# Toward eco-friendly menstrual products: a comparative life cycle assessment of sanitary pads made from bamboo pulp vs. a conventional one

**DOI:** 10.1007/s11356-025-36269-8

**Published:** 2025-03-18

**Authors:** Azita Mirzaie, Miguel Brandão, Hamid Zarrabi

**Affiliations:** 1 Department of Sustainable Development, Environment Science & Engineering, KTH Royal Institute of Technology, Stockholm, Sweden; 2 KTH Royal Institute of Technology, Stockholm, Sweden; 3 Faculty of Civil Engineering, K. N. Toosi University of Technology, Tehran, Iran

**Keywords:** Bamboo plant, Bleached wood pulp, Disposal sanitary pads, Environmental impacts, Life cycle assessment, Non-biodegradable plastics

## Abstract

The predominant menstrual products utilized by women are sanitary pads. The majority of disposable sanitary pads are comprised of plastics and bleached wood pulp, often accounting for as much as 90% of their composition by weight. Nevertheless, the overall environmental footprint of production to disposal of disposable sanitary pads is often overlooked due to the cultural stigma associated with menstruation and the lack of chemical information. This study aims to evaluate the potential environmental impact of the life cycle of sanitary pads and provide insights for decision-making and driving improvements in product design and production processes. It is important to note that this study does not aim to assess the health and safety of the products for consumer use. A case study was conducted at Hempur in Stockholm, Sweden, to assess the environmental impacts of a disposable pad made from bamboo pulp and polylactic plastic. The study applied life cycle assessment to compare the life cycle of Hempur disposable sanitary pads with conventional ones made of polymers and bleached wood pulp. The research findings reveal that Hempur sanitary pads made from plant-based materials have lower adverse environmental impacts than the conventional ones considered in the study. The upstream operations of Hempur sanitary pads were identified as the least environmentally friendly phase of the product due to the use of unbleached wood pulp and polylactic plastic, which together account for between 40 and 80% in every impact category, most notably water scarcity and abiotic element depletion. Hempur can improve its environmental performance by switching from coal to hydropower for electricity generation in bamboo pulp manufacturing, leading to a decrease in global warming and acidification. Finally, we recommend alternatives for PLA, and the use of bamboo in the core part of the Hempur sanitary pad should be considered and assessed.

**Graphical Abstract:**

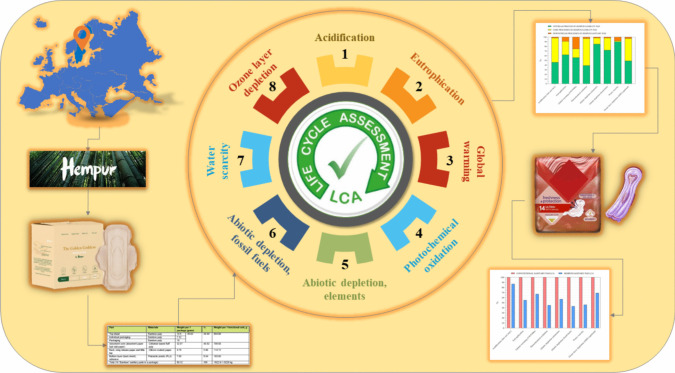

**Supplementary Information:**

The online version contains supplementary material available at 10.1007/s11356-025-36269-8.

## Introduction

In the modern world, the versatility of plastics has revolutionized the manufacturing of everyday items, replacing materials such as wood, paper, and metal due to their ease of manufacture, low cost, impermeability, and resistance to chemicals, heat, and light (Da Costa et al. 2020). One of the industries profoundly affected by this shift is the menstrual hygiene sector. Menstrual hygiene products are essential for maintaining women’s health and well-being but often have significant environmental trade-offs. Larger companies manufacture menstrual hygiene products, including tampons, menstrual cups, and sanitary pads, primarily from highly processed materials such as cotton, cellulose from wood, and non-biodegradable plastics. This reliance on disposable products stems from their convenience and profitability. However, because menstrual products are categorized as medical devices, manufacturers are not required to disclose all components or quantities. This lack of transparency contributes to an insufficient understanding of these products’ potential health and environmental impacts (Choudhary and Bhattacharjee 2018; Hait and Powers 2019).

**Fig. 1:**
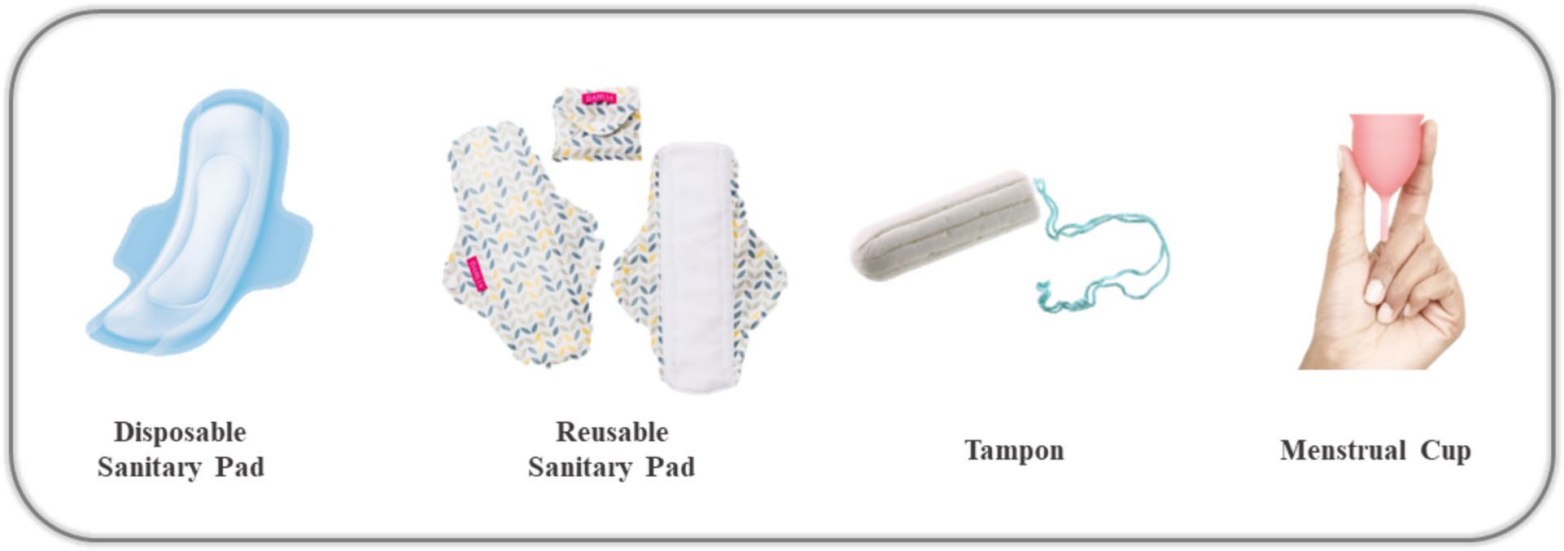
Different types of menstrual hygiene products

As shown in Fig. 1, menstruation has historically been managed with various materials and methods influenced by cultural and technological advancements (Olsson and Larsson 2014). In 1888, Johanson & Johanson introduced the first disposable sanitary pad, revolutionizing menstrual hygiene. Tampons, though invented in the early twentieth century, gained popularity only after the introduction of applicators in 1933, which addressed cultural taboos surrounding self-insertion (Hait and Powers 2019). Reusable menstrual cups, introduced in the 1930s, were not widely accepted due to material shortages during World War II and societal hesitations. Despite being a sustainable option, reusable products have struggled to compete with disposable alternatives, which became globally dominant by the mid-twentieth century. Cultural taboos and the convenience of disposables have further solidified their market position, particularly in regions with limited awareness or access to reusable options (Peberdy et al. 2019).

**Fig. 2:**
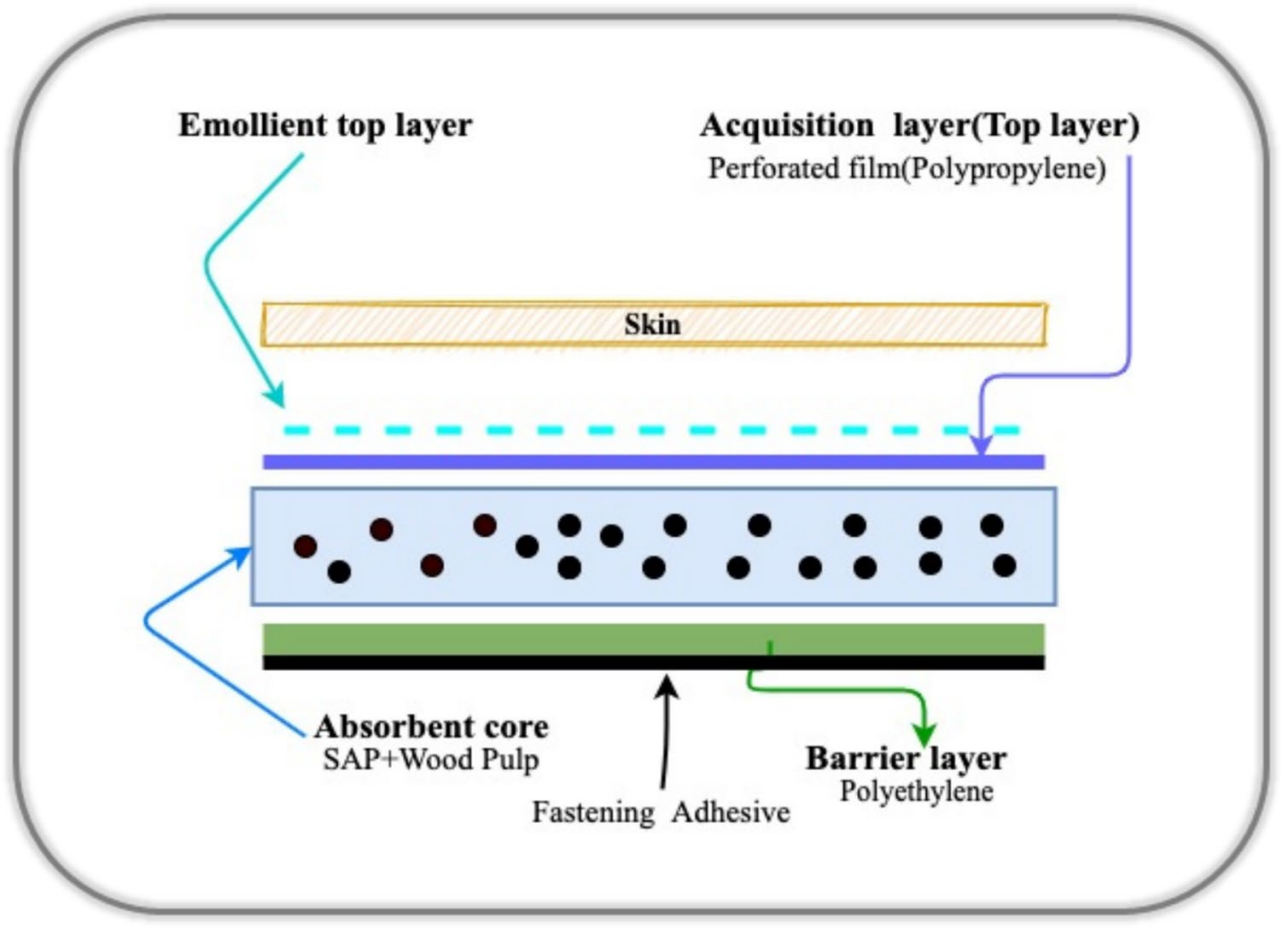
Standard structure and materials used in sanitary pads (Barman et al. 2018)

Today, disposable sanitary pads are the most widely used menstrual products worldwide. These pads cater to different needs with absorbency, shape, and size variations. As shown in Fig. 2, they typically comprise multiple layers: a top layer for fluid absorption, an absorbent core for retention, a barrier layer for leakage prevention, and an adhesive fastening system (EPD International AB 2020). The top layer, usually made from polypropylene/polyethylene fibers, ensures rapid fluid absorption and maintains dryness. Some manufacturers enhance this layer with emollients for improved comfort (Woeller and Hochwalt 2015; Li and Cogdell 2018). The absorbent core, often containing air-laid wood pulp and superabsorbent polymers (SAP), captures and retains menstrual fluid by converting it into a gel-like substance, preventing leakage. The barrier layer, typically made from polyethylene, prevents fluid from staining clothing. However, polyethylene is non-biodegradable, posing environmental challenges. The adhesive layer secures the pad to undergarments but comprises petroleum-based compounds contributing to the product’s environmental footprint (Barman et al. 2018; Li and Cogdell 2018).

Disposable menstrual products have significant environmental implications. Many sanitary pads contain up to 90% plastic and are often individually wrapped in plastic packaging. Improper disposal, such as flushing pads down toilets, exacerbates their environmental impact by clogging sewer systems and polluting aquatic ecosystems (UNEP U, 2021). Furthermore, the production and disposal of these products result in greenhouse gas emissions, resource depletion, and persistent plastic waste. These environmental challenges necessitate a comprehensive evaluation of menstrual products’ life cycles, from resource extraction (cradle) to disposal (grave). Life cycle assessment (LCA), a tool standardized by ISO 14040 and 14044, provides a framework to assess the cumulative environmental impacts of a product throughout its life cycle (ISO 2006a; Canadian Standards Association 2007).

Despite the growing awareness of the environmental burden posed by disposable menstrual products, more research is needed on sustainable alternatives. Bamboo, a rapidly renewable resource, has emerged as a potential eco-friendly material for sanitary pads. Bamboo fibers are naturally antibacterial, highly absorbent, and biodegradable, making them an attractive alternative to conventional materials (Hidayati and Iryani 2019). Bamboo pulp requires fewer processing chemicals than wood pulp, which relies on additives like polysorbate and urea-formaldehyde for bleaching and enhanced absorbency (Thilagavathi and Periyasamy 2019). Bamboo in menstrual products could reduce reliance on non-renewable resources and mitigate environmental impacts.

Several studies have evaluated the environmental performance of menstrual products using LCA. For instance, Vilabrille Paz et al. (2020) conducted an LCA in Germany, identifying reusable menstrual cups as the most sustainable option, though cultural and logistical barriers hinder their widespread adoption. Similarly, the UNEP Life Cycle Initiative highlighted the environmental benefits of reusable products, such as MakaPads in Uganda, which are locally produced and biodegradable (UNEP U, 2021). Fourcassier et al. (2022) perform a comparative life cycle assessment demonstrating that organic tampons and pads have a more significant environmental impact than menstrual cups and reusable pads, primarily due to the agricultural practices associated with organic cotton production. Harrison and Tyson (2023) and Khorsand et al. (2023) emphasize the need for sustainable menstrual hygiene solutions and policies that advance environmental sustainability and health equity, ensuring all menstruators access safe, affordable, and eco-friendly menstrual products. However, research on disposable alternatives, particularly those made from bio-based materials like bamboo pulp, remains scarce. Exploring the environmental performance of such alternatives is crucial to addressing the need for sustainable and convenient menstrual products.

Hempur, a Swedish company headquartered in Stockholm, is pioneering the development of plant-based menstrual products. Although their bamboo-based sanitary pads are manufactured in China, the company’s focus on sustainability extends beyond product design to include community empowerment and environmental stewardship. Hempur’s pads are designed to minimize environmental impact while maintaining functionality and comfort. Despite these innovations, studies have yet to comprehensively evaluate the environmental performance of bamboo-based pads compared to conventional options.

This study addresses the research gap by conducting a comparative life cycle assessment of Hempur’s bamboo-based sanitary pads and a conventional disposable model available in the European market. Focusing on Hempur sanitary pads as a case study, the research analyzes environmental impacts across the product life cycle, providing valuable insights into the potential of bamboo-based materials as a sustainable alternative to traditional menstrual products. Moreover, the study underscores the importance of incorporating eco-friendly materials into menstrual product design, contributing to the broader discourse on sustainable innovation in the hygiene industry. While centered on Hempur products, the methodology and findings are designed to be generalizable, offering insights into the environmental performance of other biodegradable hygiene products compared to conventional alternatives.

## Material and methods

In this study, the life cycle assessment method is carried out following the Product Category Rules (PCRs) for absorbent hygiene products (absorbent hygiene products are a subset of UN CPC/division 32/subclass 32193 products). The PCR specifies the rules and guidelines and defines minimum requirements for developing and communicating LCA for a specific group (EPD International AB 2020).

LCA is an internationally standardized method (ISO 2006a, b) for evaluating cumulative environmental impacts throughout a product’s life cycle. It involves collecting, calculating, and assessing inputs and outputs from various processes (Wrisberg et al. 2002; Mujkic and Andakudi Kesavan 2020; Sabour et al. 2023). LCA follows four phases: goal and scope definition, life cycle inventory analysis, life cycle impact assessment, and life cycle interpretation (Sadhukhan et al. 2014; Curran 2015). The first step, goal and scope definition, involves setting objectives, target audience, system boundaries, data quality requirements, impact categories, and study limitations (Hauschild et al. 2018).

Depending on the project’s objectives and scope, two variations of life cycle assessment are applicable: attributional and consequential LCA. In this study, an attributional LCA was employed, which involves the use of average or generic data to analyze all environmental inputs and outputs from raw material extraction to the product gate. When dealing with multifunctional processes, various allocation procedures are used to distribute environmental loads among co-products and functions (Finnveden et al. 2009; Mujkic and Andakudi Kesavan 2020). In the subsequent phase, life cycle inventory analysis (LCI), data is collected based on functional units, utilizing primary on-site data or secondary data from existing sources (Curran 2015). Finally, in the life cycle impact assessment (LCIA), LCI results are correlated with predefined impact categories, and their environmental consequences are analyzed. The last stage, life cycle interpretation, interprets and discusses LCIA results to provide recommendations (Klöpffer 2014; Curran 2015; Mujkic and Andakudi Kesavan 2020).

### Goal

**Fig. 3:**
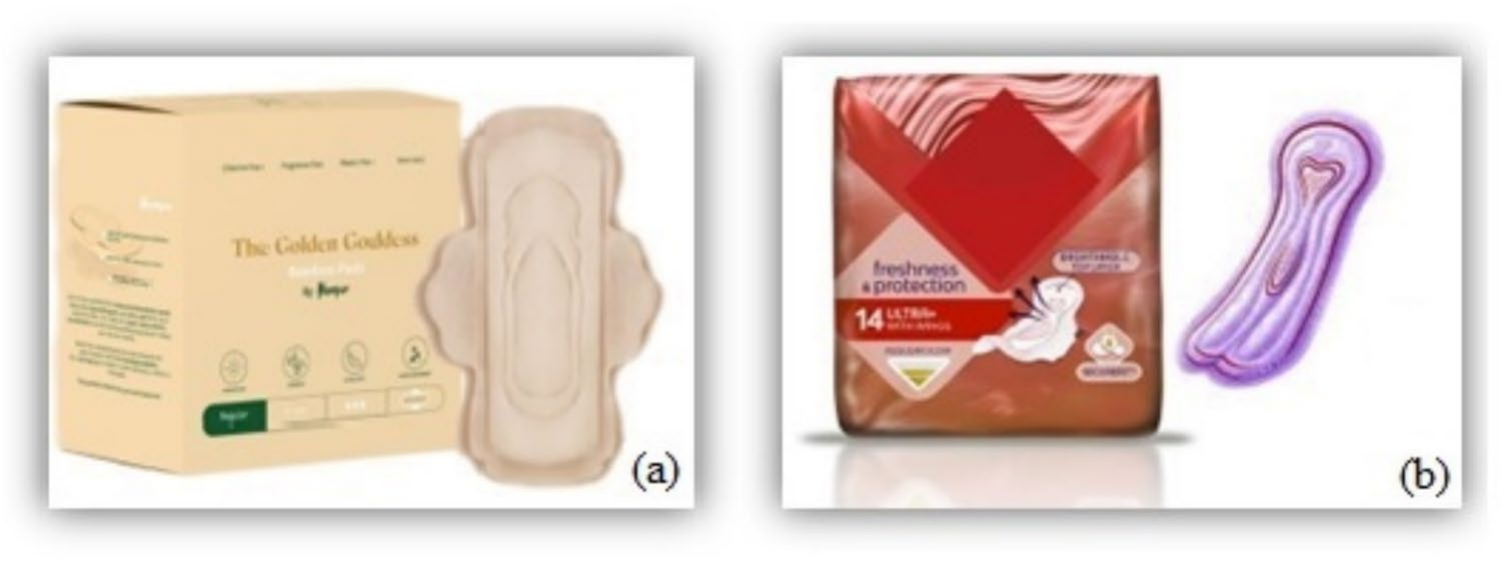
**a** Hempur plant-based sanitary pad. **b** Selected conventional sanitary pad

The current study aims to quantify and analyze the potential environmental impacts of the Hempur sanitary pad using an attributional life cycle assessment (ALCA) based on foreground primary data and background data (obtained through SimaPro software databases). While the study examines the environmental impacts of Hempur sanitary pads, the broader objective is to explore overarching trends in life cycle assessment for sanitary pads. The findings aim to identify strategies to improve the environmental performance of biodegradable hygiene products and contribute to the broader understanding of sustainable product design in the hygiene industry. After that, the results are compared with those of a conventional sanitary pad model. The following two sanitary pad models have been chosen for life cycle comparison, as shown in Fig. 3:

- Hempur sanitary pad: the plant-based and biodegradable sanitary pad, hypoallergenic, and ultra-soft (each pack contains 10 pads). These pads are made of unbleached bamboo pulp in the top sheet and unbleached wood pulp in the core part and its packaging and do not contain any fossil-based plastics.
- Conventional sanitary pad: ultra-thin sanitary pad with wings, Airtech, and SecureFit technology (each pack contains 14 pads). These pads are made of bleached wood pulp in the top and core parts, and their packaging is made of LDPE. A report by Brand Eye Research in September 2020 revealed that the most commonly used and recommended pad among women in Sweden is the ultra-thin pad with wings.

It is important to note that both models chosen for the study are aimed at the same consumer group (women between the ages of 11 and 55 who use sanitary pads as their primary menstrual product and are interested in sustainable and hygienic options).

The study aims to achieve the following objectives: (1) to identify the life cycle stages, materials, and processes that have the highest impact on the environmental performance of each sanitary pad model, (2) to determine which sanitary pad model has the best environmental performance, and (3) to develop effective strategies and recommendations to improve the performance of Hempur sanitary pads and raise awareness among women to use more eco-friendly products. The study targets several groups, including the Hempur Company, everyone involved in the production of feminine hygiene products, female customers, and researchers interested in LCA case studies or the unique supply chain and materials used by Hempur.

### System boundaries

**Fig. 4:**
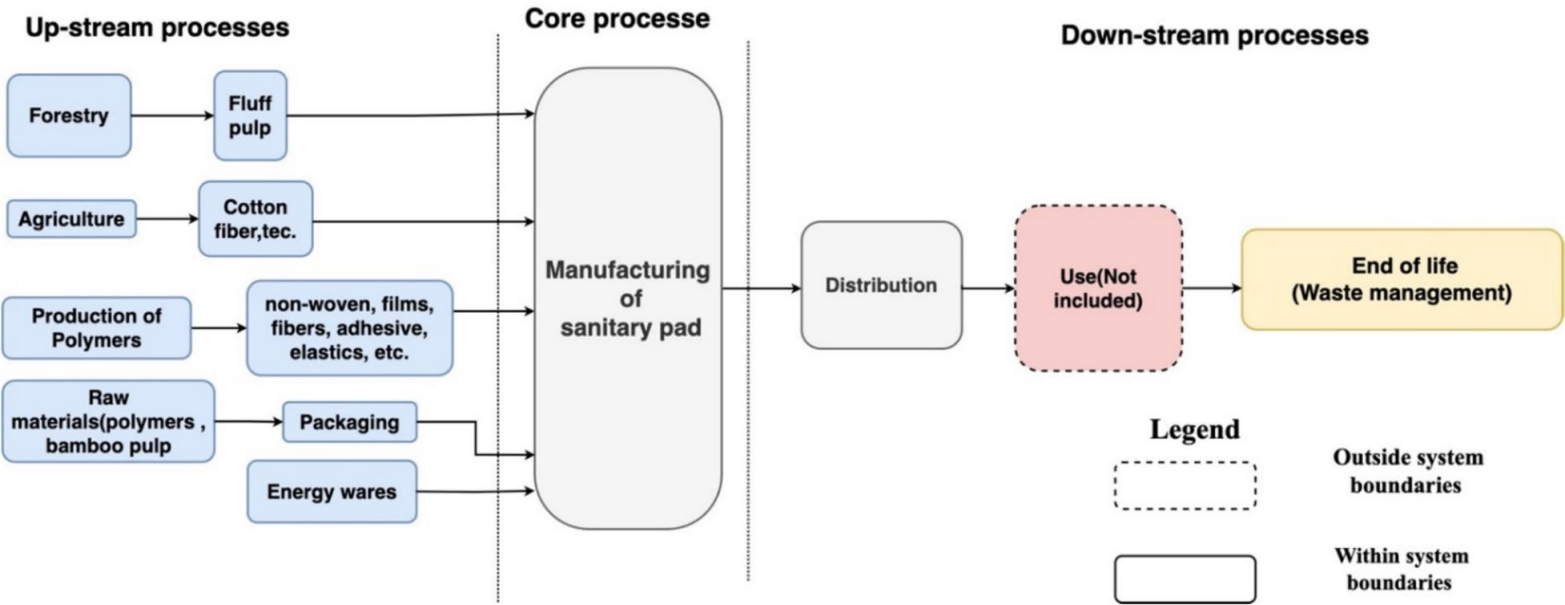
System boundaries of disposable sanitary pad (cradle-to-grave)

In this study, the system boundary is determined by the PCR for absorbent hygiene products and the EPD. The International EPD® System is a certification program for environmental declarations of type III that was established in compliance with ISO 14025—environmental labeling and declarations (EPD International AB 2020). Accordingly, the system boundary considers the entire life cycle of the product from resource extraction to the final disposal (cradle-to-grave). Disposable products, like sanitary pads, are meant to be used only once. For example, a disposable sanitary pad can be used for 4–8 h and must be thrown away immediately after use. Therefore, it is not important to focus on their usage phase. According to Fig. 4, the life cycle of a sanitary pad is divided into three stages:

1. Upstream processes (cradle-to-gate): The following processes are considered to be upstream processes used for the manufacture of sanitary pads:
2. Extraction and refinement of natural resources (e.g., forestry, agriculture, and extraction of oil)
3. Production of packaging materials, excluding pallets
4. Production of raw materials (e.g., pulp, cotton and other fibers, non-woven, laminates, superabsorbent, adhesive)
5. Production processes of energy wares used for upstream processes
6. Core processes (gate-to-gate): These processes are classified to
7. Transportation of input materials to the core process
8. Manufacturing of sanitary pad
9. Production of energy wares used for core processes
10. Downstream processes (gate-to-grave): These processes are as follows:
11. Transportation from final manufacturing to average customers or distribution point
12. Waste management of used sanitary pads (packaging included)

It is important to note that the geographic scope of this study is Sweden, which has advanced energy mixes and waste management systems. This focus may limit the generalizability of the findings to other regions with different energy sources or waste management infrastructures. Specifically, regions with coal-based energy systems or less efficient waste management systems could yield different environmental impact profiles for the Hempur and conventional sanitary pads. Furthermore, Sweden’s advanced waste management systems, which favor the incineration or composting of waste, might lead to more favorable environmental performance for the Hempur sanitary pads. In regions where such systems are less prevalent, the environmental impacts of the Hempur pad, particularly regarding waste management, may differ. This study assumes the typical Swedish waste management practices and energy mix, which may not apply to all regions.

### Cut-off rules

Due to the disposable feature of sanitary pads, the use phase is cut off. All raw materials involved in producing each product part are accounted for. A minimum of 99% of the total weight of the declared product (including its packaging) is included. The technical system will not involve the production of manufacturing equipment, construction, or other capital goods. The analysis will not include the pallets or cardboard boxes used for transporting the product. Finally, it was assumed that the downstream processes take place in Stockholm.

### Functional unit (FU)

Due to their single-use nature, women use a considerable amount of disposable sanitary pads over the course of their lives. The functional unit is defined as 1 year of coverage for one woman. The following consumption patterns are included in the functional unit:

- The number of units used each day (sanitary pads provide coverage for 4 to 8 h)
- Lengths of the menstrual cycle (usually 3–7 days, although can vary from woman to woman)

Thus, 108–504 items are required to cover a woman’s menstrual demands for a year. According to other studies on the use of feminine care products, women typically use 240 disposable commercial sanitary pads annually (Kane 2015; Hait and Powers 2019). Therefore, 240 commercial disposable sanitary pads served as the functional unit for this study. Consequently, 17 packages of the conventional sanitary pad and 24 packages of the Hempur sanitary pad are needed to absorb menstrual fluid within a year, which is how the reference flow is determined.

### Data collection

A qualitative and quantitative investigation is carried out for two selected models to comprehend the production processes, materials utilized in sanitary pad manufacture, and their compositions. This paper included a comprehensive review of the literature, interviews with sanitary pad producers, and input from other sanitary pad industry specialists.

Specific data on the Hempur (plant-based) sanitary pad’s materials and production processes were obtained directly from Hempur Company experts and their suppliers. This includes details on bamboo cultivation, pulp manufacturing, and other material and energy requirements specific to their production processes. However, inputs for the conventional sanitary pad model, such as material compositions, raw material processing, energy requirements, and transportation, were derived from the most recent sustainability report of a leading brand in the industry (the name of the report cannot be disclosed due to confidentiality agreements). While this data source is reliable, we acknowledge that it may not fully account for all production variables or provide complete transparency, as noted in the “Limitations and uncertainties” section.

Uniform transportation and energy scenarios were modeled wherever possible to ensure consistency in the comparison between the Hempur and conventional pads. Specifically, the regional energy mix for Sweden was applied consistently across both product systems, and average European transport distances were used for transporting raw materials and finished products to ensure a balanced analysis. When specific transportation details were unavailable, generic assumptions were based on Ecoinvent 3.10 data, ensuring alignment with recognized LCA data standards.

Following the Product Category Rules for absorbent hygiene products, data on the system under consideration, including material and energy flows, were collected from primary sources wherever possible. Primary data for the Hempur pads were gathered directly from the company’s sites and contractors. In cases where specific data were unavailable, selected generic data from established commercial databases (e.g., Ecoinvent 3.10) and accessible databases meeting data quality requirements were used. If neither specific nor generic data were available, proxy data were employed, and a detailed rationale for their selection was documented to ensure transparency.

The product systems under examination were modeled using SimaPro (version 9.2.0), which generated results through characterization models and impact assessment methods. Regional energy mixes and transportation flows were sourced from the Ecoinvent 3.10 database, while waste statistics were derived from the most up-to-date OECD database. The geographic scope of the study is Sweden, and end-of-life scenarios were aligned with current regulations and practices in the region, focusing primarily on incineration due to its prevalence.

### Impact categories and impact assessment methods

Life cycle impact assessment refers to the stage that seeks to comprehend and appraise the extent and import of the probable environmental consequences of a product system. In consonance with the objectives of the present study and in compliance with the absorbent hygiene products PCR guidelines, the European EPD (2018) impact assessment methodology was employed to scrutinize the seven impact categories delineated in Table 1. The EPD International AB (2020) approach represents a progression from its predecessor, EPD (2013), and serves the purpose of formulating Environmental Product Declarations (EPDs). This is detailed on the website of the Swedish Environmental Management Council (SEMC). The most recent revision to the guidelines incorporated within this approach dates back to June 8, 2018 (encompassing the integration of the water scarcity footprint). The majority of impact categories are derived directly from the CML-IA baseline technique, encompassing eutrophication, global warming, ozone depletion, and abiotic resource depletion. Moreover, the CML-IA non-baseline method, specifically acidification, is also included in the impact categories. The categorization of water scarcity is established through the utilization of the AWARE methodology, whereas the classification of photochemical oxidation is predicated upon the ReCiPe 2008 framework. All of these distinct methods can be found within the SimaPro software.

**Table 1:** Impact categories and assessment methods used in this study

Impact category	LCIA method
Global warming	CML-IA baseline v4.1/EU25
Eutrophication	CML-IA baseline method, v4.1/EU25
Acidification	CML-IA non-baseline v4.1/EU25
Formation of tropospheric ozone	ReCiPe 2008
Depletion of abiotic resources—elements	CML-IA baseline v4.1/EU25
Depletion of abiotic resources—fossil fuels	CML-IA baseline v4.1/EU25
Water scarcity	AWARE method

## Results and discussion

### Life cycle inventory (LCI) analysis

This section explains the assumptions, data collection, and calculations used to create the LCI of two models of sanitary pads.

#### Hempur sanitary pad life cycle inventory

**Fig. 5:**
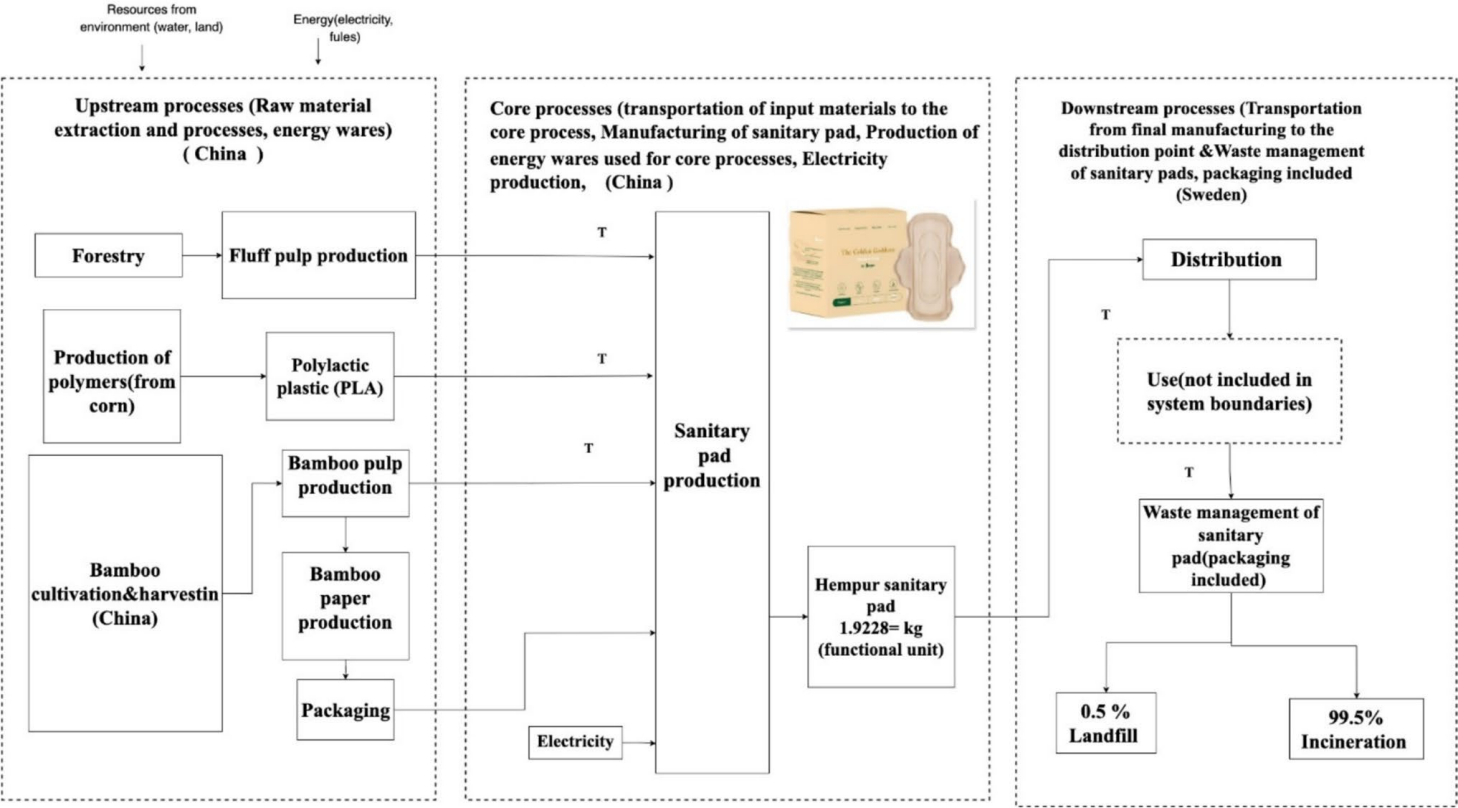
Process flow chart of Hempur sanitary pad

The various processes employed in creating the LCI model of the Hempur sanitary pad are illustrated in Fig. 5.

##### Raw materials

The list of all constituent elements evaluated for Hempur’s sanitary pads is presented in Table 2. The data about these measurements have been meticulously gathered by Hempur’s proficient experts and subsequently converted into the functional unit.

**Table 2:** Assemblies and subassemblies of the bamboo sanitary pad of Hempur Company

Part	Materials	Weight per 1 package (gram)	%	Weight per 1 functional unit, (gram)
Top sheet	Bamboo pulp	10.5	13.1	251.98
Individual packaging	Bamboo pulp	7.12	8.89	170.87
Packaging	Bamboo pulp	18	22.47	431.98
Absorbent core (absorbent paper & air-laid paper)	Unbleached wood pulp	32.07	40.02	769.65
Back, wing release paper, and little tap	Paper (silicon coated)	4.78	5.97	114.72
Bottom layer (back sheet), adhesive	Polylactic plastic (PLA)	7.65	9.55	183.6
Total (10 bamboo sanitary pads in a package)		80.12	100	1922.8

##### Transportation

Hempur’s transportation endeavors primarily center around the conveyance of unprocessed materials to manufacturing facilities and the transportation of finalized merchandise to their respective patrons. According to the data presented by the Hempur Company, the cultivation of bamboo and the subsequent pulping process occur in the province of Guizhou, specifically in the region of Chishui. This location is situated approximately 1100 km away from the coast. Following processing, bamboo commodities are transported nearly 180 km to Chongqing Port situated on the Yangtse River, from where they are subsequently conveyed by riverboat to the Hong Kong Port, resulting in a voyage of roughly 3572 km, intended for transport to destinations around the globe. The present study examines the circumstance in which Hempur sanitary pads are transported from the port of Hong Kong, China, to the port of Gothenburg, Sweden, via ship, encompassing a distance of approximately 18,875 km, passing through the Suez Canal. Subsequently, the sanitary pads are shipped by truck for roughly 170 km to the Hempur Company warehouse as the final destination located in Värnamo in Sweden. The transportation used corresponds to each functional unit for each distance will be as follows:

- Local transport of products by truck from Guizhou Province to the Chongqing Port in China is 0.34 tkm.
- River transportation from Chongqing Port to Shanghai Port by riverboat is 6.86 tkm.
- Shipping from Shanghai Port to the port of Gothenburg, Sweden, by ship is 36.29 tkm.
- Local transport of product to the destination in Sweden by truck is 0.32 tkm.

##### Energy consumption

The production of a single ton of bamboo pulp requires 2 t of bamboo plant, as well as 100 kg of ethanol and 100 l of water. Concerning the utilization of energy for the production of every ton of bamboo pulp, a total of 519 kWh of electricity produced by hard coal and 1474 kWh of steam are required. The Hempur Company’s yearly production of bamboo-based sanitary pads amounts to a total of 24 million packages. The annual production necessitates an electrical energy consumption of 600,000 kWh, resulting in an energy requirement of 2.5 Wh to manufacture a single Hempur sanitary pad. Therefore, the electricity energy requirement corresponding to a functional unit is 0.6 kWh.

##### Waste management

The downstream processes associated with the Hempur sanitary pads are situated in Stockholm, Sweden. Disposable sanitary pads are classified as household waste by the Swedish waste management system, with 0.5% of household waste ending up in landfills. Waste incineration constitutes a sanitary and ecologically sound manner for eliminating sanitary pad waste that is not amenable to recycling. The present LCA analysis assumes that a majority, that is, 99.5%, of the sanitary pads undergo incineration at the Högdalenverket incineration plant located in Stockholm, while a minor proportion (0.5%) is disposed of at the Sofielund landfill plant, also situated in Stockholm.

#### Conventional sanitary pad life cycle inventory

**Fig. 6:**
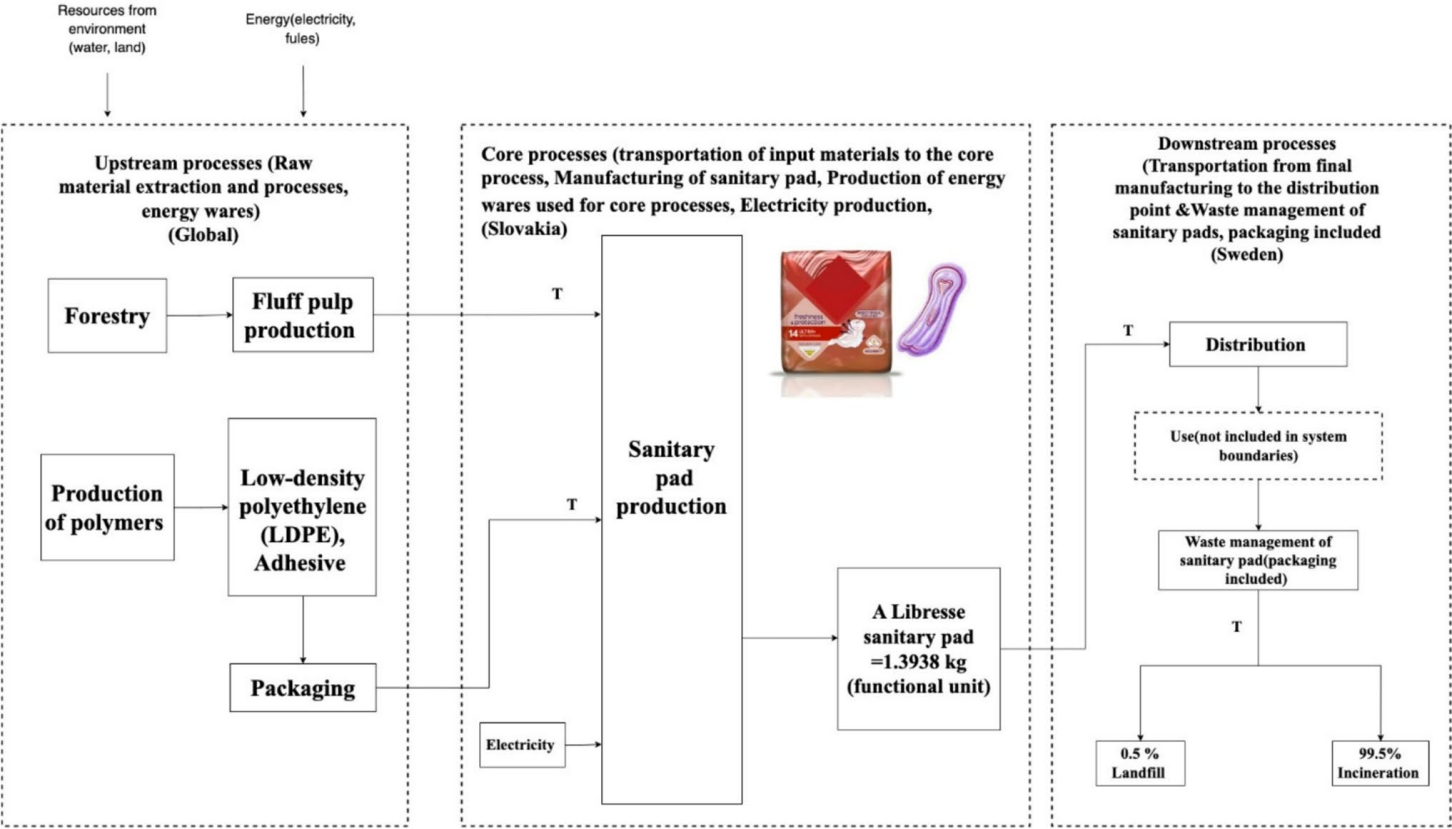
Process flow chart of conventional sanitary pad model

The entirety of the processes for the conventional sanitary pads are illustrated in Fig. 6.

##### Raw materials

All constituents of conventional sanitary pad models are itemized in Table 3. The data for these measurements were collected by experimentally weighing sanitary pads, with an error margin of 0.0001 g, and subsequently converting them into a singular functional unit.

**Table 3:** Assemblies and subassemblies of conventional sanitary pad model

Part	Materials	Weight per 1 package (gram)	%	Weight per 1 functional unit (gram)
Absorbent core (pad filling)	Bleached wood pulp	52.02	63.45	88.44
Paper sheet	Paper (silicon coated)	1.70	2.07	28.8
Bottom layer (back sheet)	Low-density polyethylene (LDPE)	13.78	16.8	234.2
Individual packaging	Low-density polyethylene (LDPE)	11.50	14.02	195.4
Packaging	Low-density polyethylene (LDPE)	3	3.66	51
Total (14 conventional sanitary pads in a package)		82	100	1393.8

##### Transportation

Typically, a conventional sanitary pad company’s transportation activities consist of transporting raw materials to its manufacturing facilities and delivering finished products to consumers. The company procures transportation services from third parties. The majority of the company’s incoming items are transported by sea, while the majority of its outbound products are transported by road and rail. The total transportation usage of the conventional sanitary pad manufacturer is 13 billion tkm or 4.94 tkm per functional unit. The distribution of transportation modes is 58% sea freight, 38% truck, and 4% rail. Consequently, the transport allocation will be as follows: Ship—28635 tkm, truck—18760 tkm, and rail—0.1974 tkm.

##### Energy consumption

Energy for the production process is purchased from the national grid, and biofuels and fossil fuels are supplied to the production site. Table 4 indicates the total amount of different types of energy used in total production and per functional unit.

**Table 4:** The total amount of energy consumption per functional unit

Energy utilization	Total amount (GWh)	Per functional unit (kWh)
Purchased electricity	4231	1.61
Purchased heating/steam	181	0.07
Biofuel	1129	0.43
Fossil fuel	6919	2.63
Total fuels	8048	3.06
Total energy	12460	4.73

##### Waste management

In 2020, conventional sanitary pad manufacturers managed their production waste in a variety of methods. The sanitary pads were sent to municipality waste facilities where the used products are incinerated or sent to landfill. A total of 99.5% of sanitary pads are incinerated in the Högdalenverket incineration plant in Stockholm, and 0.5% of them go to the Sofielund landfill plant in Stockholm.

### Life cycle impact assessment (LCIA)

#### Hempur sanitary pad life cycle impact assessment

**Fig. 7:**
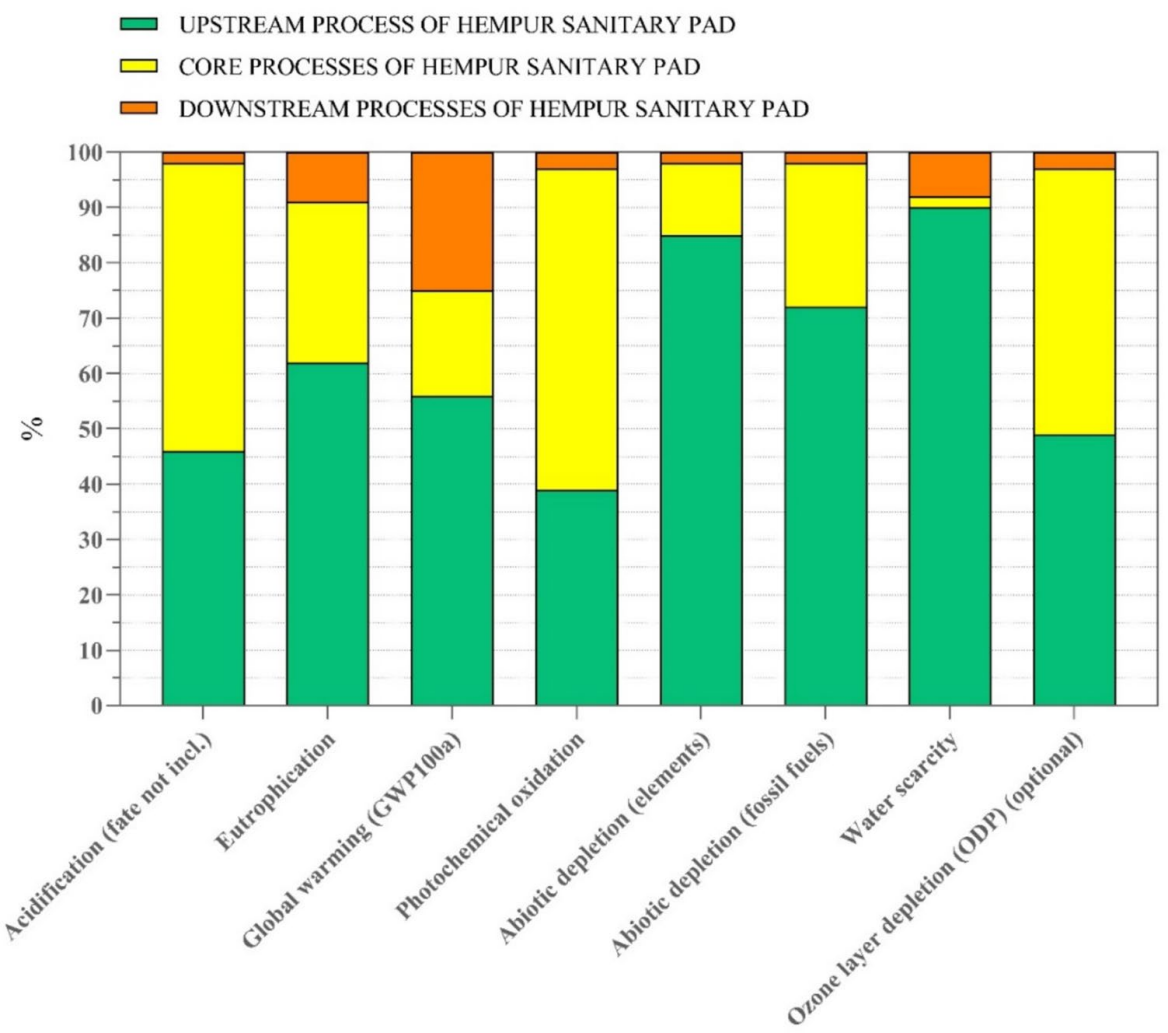
Contribution analysis of Hempur sanitary pad’s life cycle stages

Based on the findings in Fig. 7, the assessment of Hempur sanitary pads indicates that the upstream and core processes have the greatest impact on the pad’s life cycle. Despite the use of eco-friendly materials, the upstream processes still significantly contribute to the pad’s environmental performance, with approximately 90% contribution to water scarcity. To further investigate the environmental performance of Hempur sanitary pads, the aspects and biggest contributors will be analyzed. The supplementary material: Table 1 contains the potential environmental impact of Hempur sanitary pads by the life cycle stage.

##### Acidification

In the impact category of acidification, over the life cycle of Hempur, approximately 0.024 kg SO_2_ eq is emitted. The core processes and upstream processes contribute almost equally to this impact, at 52% and 46%, respectively. The downstream phase, on the other hand, only accounts for a minor contribution of 2%. During the core processes, the transportation of sanitary pads from Shanghai Port in China to Gutenberg Port in Sweden via Transocean ship is responsible for 74% of the emissions released during the core phase of the product life cycle. This is followed by inland waterway transportation, which accounts for 23% of the emissions. Polylactide granulate used in the Hempur pad is responsible for 27% of the emissions released during the upstream phase of the product life cycle. Additionally, electricity production from hard coal is responsible for 24% of the total impact, while heat from steam in the chemical industry and unbleached sulfate pulp account for 14.7% and 15.1%, respectively, in this phase.

##### Eutrophication

The eutrophication impact category shows that 0.0059 kg of PO_4_ eq is emitted per functional unit. The upstream processes contribute 61.8%, the core processes contribute 28.7%, and the downstream processes contribute 9.48%. Similarly, in the acidification impact category, transportation by transoceanic ship from China to Sweden contributes 59% of the core processes emissions, while inland transportation by ship in China waterways accounts for 35.9%. Polylactide granulate accounts for 40.2% of the upstream processes’ emissions, while printed paper and unbleached sulfate pulp contribute to 15.5% and 13.2%, respectively. Lastly, the downstream processes are responsible for emissions during the municipal solid waste incineration treatment, while the landfill of municipal solid waste with gas utilization and leachate treatment has an adverse impact of 4.41%.

##### Global warming

Throughout the life cycle of Hempur sanitary pads, approximately 3.98 kg of CO_2_ eq emissions are released. Of this aggregate, 55.8% of the pad’s emissions are attributed to the upstream processes, 19.5% to the core processes, and 24.7% to the downstream processes. The discharge of CO_2_ eq emissions during the downstream processes (0.98 kg) is solely associated with waste incineration. The primary contributor to global warming is linked to the treatment of municipal solid waste incineration, which accounts for 24.7% of the total CO_2_ eq emissions during the entire life cycle of Hempur sanitary pads. The transportation of the pads from China to Sweden, via transoceanic ship, accounts for 43.5% of all emissions in the core processes. The upstream processes generate the majority of total emissions, with polylactic granulate responsible for 25.2% of emissions. Electricity production from hard coal and heat from steam in chemical industries make up the same number of emissions, each contributing 23% to the total impact during this phase. Printed paper and unbleached sulfate pulp are the next highest contributors to the pad’s global warming impact, accounting for 13.2% and 8.6%, respectively.

##### Photochemical oxidation

During the three stages of the Hempur sanitary pads’ life cycle, it is estimated that 0.021 kg NMVOC eq emissions is released into the air. The majority of these emissions come from the core (57.7%) and upstream (39.2%) processes, with a small contribution from the downstream phase (3.03%). The transportation of sanitary pads from China to Sweden by transoceanic ships alone accounts for 64% of total emissions released during the core stage of the product life cycle; the remained emissions come from other modes of transportation, including inland waterway transportation by riverboat and on land by lorry. Throughout the upstream processes, the emission of polylactic material and unbleached sulfate pulp accounts for 26.1% and 22.4% of the total emissions, respectively. Additionally, other factors that contribute to environmental performance in this phase include the production of electricity from hard coal (20.2%), printed paper (12.3%), and the use of heat from steam in the chemical industry (11%). Lastly, the total emissions in the downstream stage are attributed to waste incineration.

##### Abiotic depletion, elements

Throughout the entire life cycle of the Hempur sanitary pad, a total of approximately 1.1 × 10^−5^ kg Sb eq is utilized. This impact is primarily generated in the upstream phase, accounting for 85.2% of the overall consumption, while the core processes contribute 13.2%, and the downstream stage is responsible for 1.54%. Notably, the polylactic granulate is the foremost contributor to the uses in the upstream processes, accounting for 55.2%, followed by unbleached sulfate pulp at 16.3%, printed paper at 13.5%, and ethanol at 12.2%. The core processes are predominantly impacted by transportation, with inland waterway transportation via ship accounting for 38.6% and global transportation via lorry contributing 28.6%. Lastly, municipal solid waste incineration is primarily responsible for the overall impact of the downstream processes.

##### Abiotic depletion, fossil fuels

Throughout its life cycle, the Hempur sanitary pad is accountable for the consumption of approximately 37.7 MJ of fossil fuels, which are utilized to provide the necessary energy for the production of materials such as plastics. It is noteworthy that the majority of this consumption, specifically 71.9%, is related to the upstream phase, whereas the core processes account for 26.6%, and the downstream phase contributes only 1.56%. Among the materials employed in the production of the Hempur sanitary pad, polylactic granulate is responsible for the highest amount of emissions, totaling 24% of the life cycle’s total emissions. Additionally, the heat generated from steam is accountable for 23.2%, while electricity production from hard coal contributes 16.7%. Printed paper is responsible for 13.5% of the emissions, while ethanol and unbleached sulfate pulp account for 14.2% and 8.43%, respectively. Along the core processes, the total emissions are generated alone by transportation. The transportation of sanitary pads by ships is responsible for 83.1% of the total impact, and transportation by lorry accounts for 16.74%. Additionally, in the down processes, 100% of the consumption takes place during the household waste treatment by incineration.

##### Water scarcity

Throughout the life cycle of Hempur sanitary pads, a significant consumption of water amounting to 1.92 m^3^ is estimated. The upstream processes, which account for a noteworthy 89.9%, require a substantial quantity of water. On the other hand, the downstream processes only contribute 8.3% of emissions, while the core processes represent a mere 1.76% of the total impact in this stage. Although the polylactic granulate utilized in Hempur sanitary pads is responsible for 46.5% of the total impact, other materials such as unbleached sulfate pulp (36%) and printed paper (10.3%) also contribute to the upstream results. Furthermore, the transportation of sanitary pads plays a significant role in the core processes, with 81.3% of the total impact attributed to transportation by ships and 15.7% to transportation by lorries.

##### Ozone layer depletion

Lastly, concerning the impact category of ozone depletion, the Hempur sanitary pad emits a total of 2.5 × 10^−7^ kg CFC-11 equivalent during its life cycle. The upstream processes are responsible for 49.6% of this total and the core processes for 47.7%, whereas the downstream processes account for 2.68% of total emissions. In the upstream phase, the emissions discharged from the heat from steam in the chemical industry represent 33.3 of the total. The other components that contribute to the upstream emissions are the polylactic granulate (29.2%) and unbleached sulfate pulp (21%). Additionally, the contributors to the emissions produced in the core processes are transportation by ship 83.2% and transportation by lorry 16.7%.

#### Conventional sanitary pad life cycle impact assessment

**Fig. 8:**
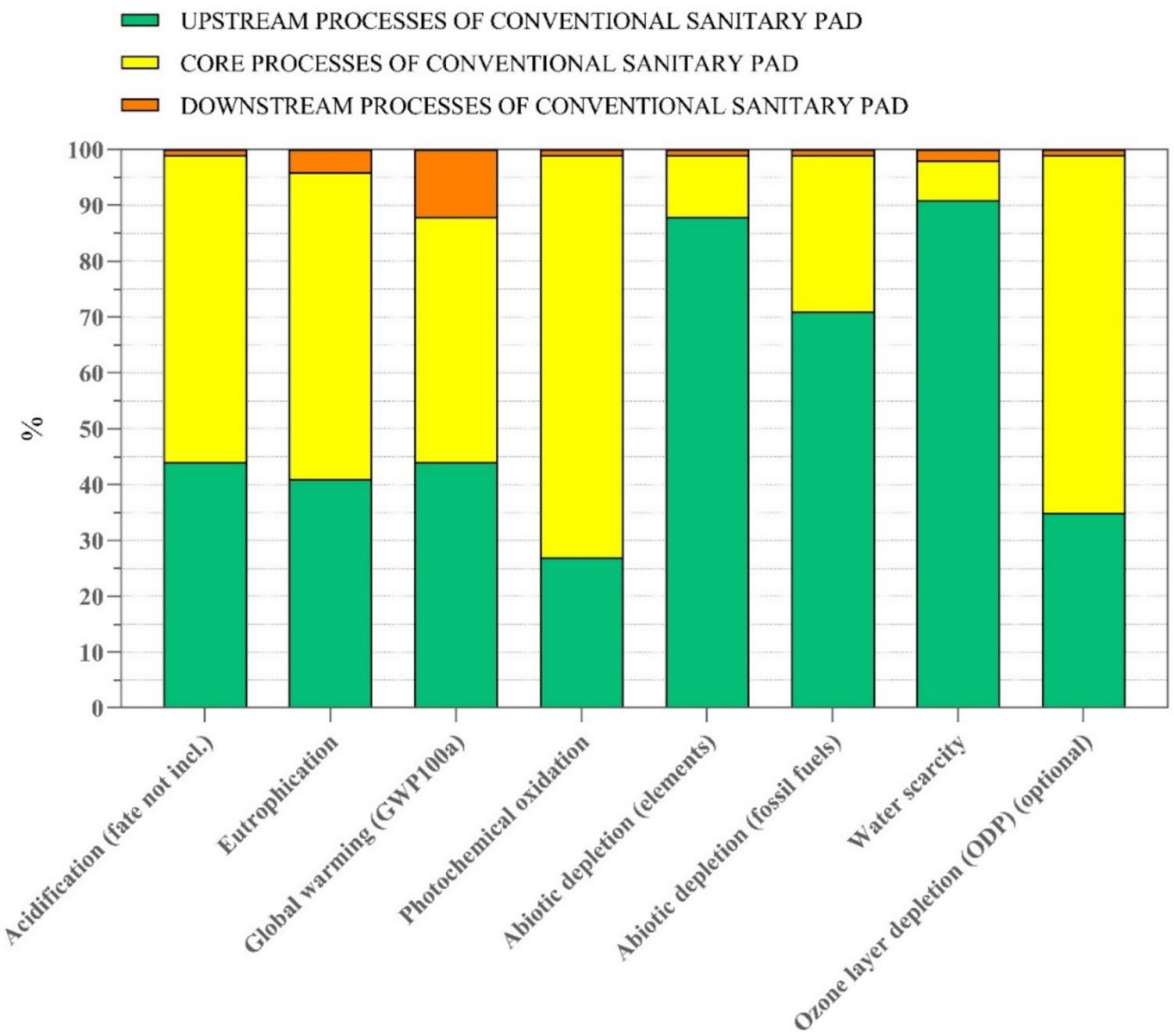
Contribution analysis of conventional sanitary pad’s life cycle stages

As illustrated in Fig. 8 and following the outcomes of the Hempur sanitary pad’s impact assessment, the foremost determinant of the conventional sanitary pad’s environmental performance lies in its upstream and core processes. In the three assessed categories of water scarcity, abiotic depletion of fossil fuels, and abiotic depletion of elements, the upstream process accounts for more than 50% of the total impact. Additionally, in the impact categories of ozone layer depletion, eutrophication, and photochemical oxidation, the core process contributes to more than 50% of the total impact. In the following sections, the determining factors and major contributors to the conventional sanitary pad model’s environmental performance are analyzed in terms of hotspots in each impact category. The supplementary material: Table 2 contains the potential environmental impact of conventional sanitary pads by the life cycle stage.

##### Acidification

Throughout the life cycle, it has been approximated that the conventional sanitary pad design discharges a sum of 0.03 kg SO2 eq. The upstream processes are accountable for 43.6%, and the core processes are responsible for 55%, with a marginal contributor from downstream processes (1.39). Over half of the impact from upstream processes originates from the packaging film and sulfate-bleached pulp, which account for 66.4% and 29.5%, respectively. The printed paper has the lowest contribution to the upstream emissions (4.11%). Diesel combustion emits 53.7% through the core processes, while 30.1% is released through electricity use, and transportation accounts for 14.18%. Additionally, all emissions discharged during downstream processes come from the incineration of household waste.

##### Eutrophication

Throughout the life cycle of the conventional sanitary pad model, it is estimated that 1.1 g PO4 eq is released. The emissions are distributed as follows: the upstream processes account for 41.5%, the core processes account for 54.7%, and the downstream processes only represent 3.84%. Among the upstream processes, the packaging film and sulfate-bleached pulp contribute the most to the environmental impact, with 61.7% and 33.8%, respectively. Additionally, printer paper accounts for a mere 4.53% of total emissions in this life cycle. Out of the total emissions released along the core processes, electricity consumption accounts for 66.7% and diesel burning for 25.0%. Finally, the incineration of the conventional sanitary pad model represents 100% of downstream emissions.

##### Global warming

Throughout its entire life cycle, the conventional sanitary pad model releases a total of 5.93 kg CO_2_ equivalent. Nearly most of the emissions are divided between the upstream (44.1%) and core process stages (43.9%). The main contributor to the upstream emissions is the production system of the back sheet made of packaging film (79.8%). Other great contributors are sulfate-bleached pulp (16.3%) and printed paper (3.95). Moreover, in the core processes, electricity use accounts for 31.5%, diesel burning is responsible for 32.9%, and the core process of the conventional sanitary pad model accounts for 20.8% of total emissions in this life cycle due to the extraction of water from nature and emissions to air and water. The rest of the emissions of the core processes of the conventional sanitary pad model are generated by the transportation of materials and products. Lastly, the only contributor to the emissions produced in the downstream processes is the incineration of sanitary pad waste.

##### Photochemical oxidation

Along the three stages of the conventional sanitary pad model’s life cycle, it is estimated that 0.047 kg NMVOC is released into the air. The upstream processes are responsible for 27.3% of this total, the core processes for 71.7%, and the downstream processes for 0.9%. In the upstream process, the packaging film (LDPE) represents 68.6%, the next component sulfate-bleached pulp is the second contributor (28.6%), and another component with smaller contributions is the printed paper 2.8%. The core processes emit 0.03 kg NMVOC, in which the electricity produced by the burning biomass is responsible for 51.3% of the emissions, whereas the electricity used by the sanitary pad manufacturer contributes to 5.74%, diesel burning contributes to 35.7%, and transportation 5.1% has the smallest contribution of the impact of this phase. Additionally, in the downstream processes, 100% of emissions are released during the incineration of the sanitary pad in the waste treatment plant.

##### Abiotic depletion, elements

In the impact category of abiotic depletion elements, the conventional sanitary pad model uses roughly 1.86E−5 kg Sb eq of virgin abiotic material during its life cycle. It is estimated that upstream processes are the main consumers of abiotic elements at 88%, core processes at 11.4%, and downstream processes at 0.6%. In the upstream processes, the sulfate-bleached pulp represents 71.6% of consumption; next, packaging film (LDPE) used in the back sheet of the sanitary pad is the second biggest contributor (25.7%); and another component with a smaller contribution is printed paper (2.66%). Transportation by lorry is the main consumer of abiotic material with 52.8% of the total impact in the core processes, followed by diesel burning (16.5%) and electricity use (26.9%). Furthermore, the incineration of sanitary pad waste is only responsible for abiotic depletion in the downstream phase.

##### Abiotic depletion, fossil fuels

Throughout the conventional sanitary pad model’s life cycle, around 86.9 MJ of fossil fuel is extracted to provide energy resources. The upstream processes are the main contributor, with 70.8% of the total impact. The core processes are responsible for 28.7%. The downstream processes with only 0.4% have the smallest portion of the total impact. The back sheet of sanitary pads made of packaging film represents 89% of the total where most of the impact occurs, whereas sulfate-bleached pulp and printed paper are the minor contributors to the total impact in this stage, 8.8% and 2.1%, respectively. In the core processes, diesel burning accounts for nearly half its emissions 48%, followed by electricity use (31%) and transportation by lorry (18.4%). Moreover, in the downstream processes, the waste management, i.e., incineration of the sanitary pad, is responsible for 100% of the impact on this phase.

##### Water scarcity

A 4.12 m^3^ water is extracted throughout the conventional sanitary pad model’s life cycle. The upstream processes are responsible for the most contributors to water scarcity with 91%, whereas the core possesses (6.8%) and downstream processes (2.2%) have the smallest portions of the total impact. The sulfate-bleached pulp is responsible for more than half of the total impact in this stage (56.6%). The next materials that contribute to the conventional sanitary pad model’s environmental performance are packaging film (41.7%) and printed paper (1.68%). Additionally, in the core processes, electricity use is mainly responsible for water scarcity at 72% and diesel burning at 16%, while core processes of the conventional sanitary pad model due to the extraction of water resources and emissions to the natural water are responsible for 16% of the total impact in this life cycle phase.

##### Ozone layer depletion

Finally, considering the impact category ozone layer depletion, the conventional sanitary pad model releases during its life cycle 3.64E−7 kg CFC-11 equivalent. The upstream processes are responsible for 34.7% of this total, and the core processes are the main ones responsible for the total emissions (63.9%), while the downstream processes account for only 1.3% of total emissions. In the upstream processes, the emissions discharged from the sulfate-bleached pulp production represent 53% of the total, followed by the packaging film at 42.6%, while the printed paper accounts for 4.47% of the impact. Moreover, the contributors to the emissions produced in the core processes are diesel burning (63.6%), electricity use (8.8%), and transportation by lorry (24%). Lastly, 100% of the emissions during the downstream processes are due to the incineration of the sanitary pad waste treatment process.

#### Comparative life cycle impact assessment of two sanitary pad models

**Fig. 9:**
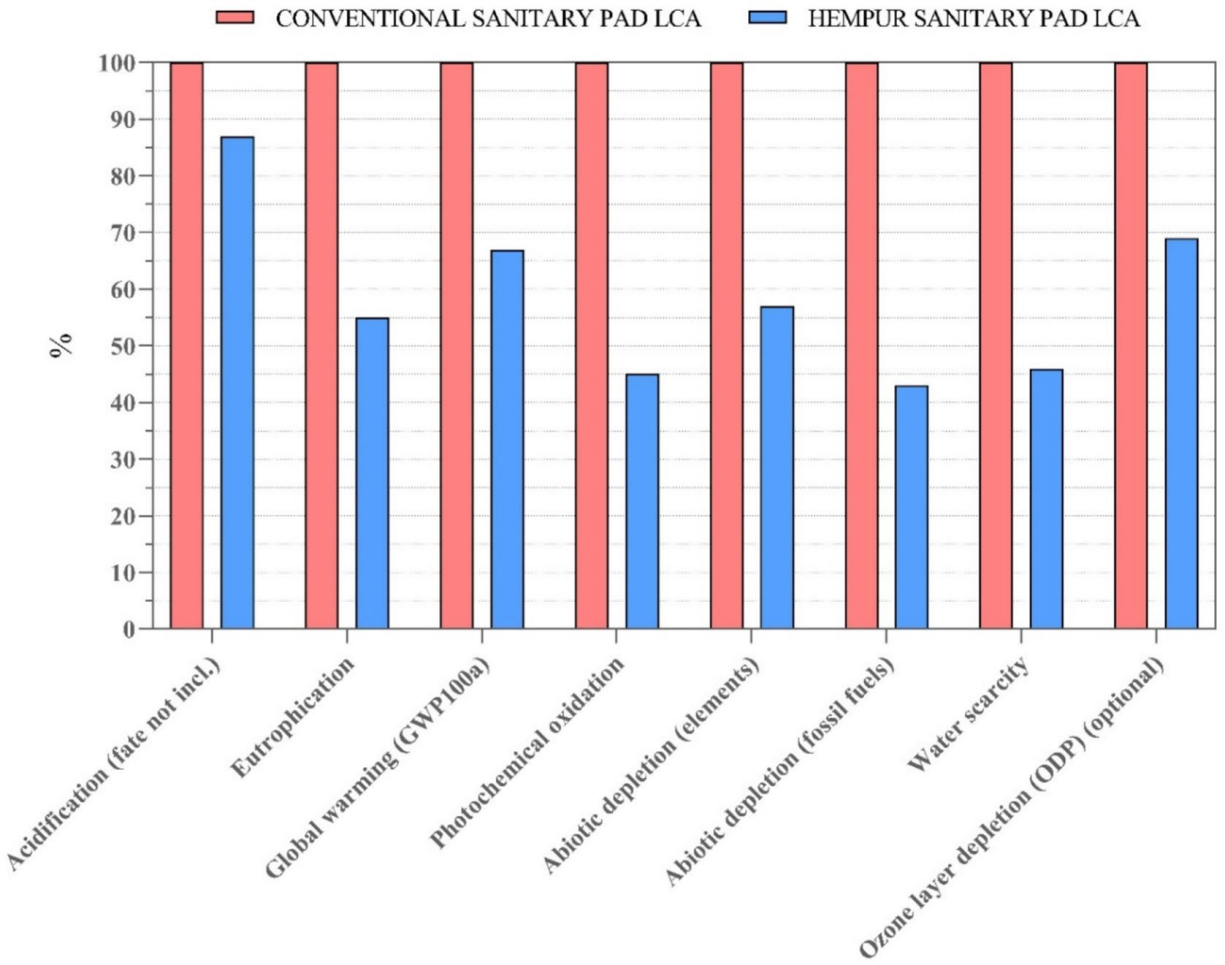
Impact assessment of the two sanitary pad models

As depicted in Fig. 9, the analysis of eight impact categories revealed that the conventional sanitary pad model exhibits the highest environmental impact across all categories assessed (in all impact categories, the Hempur sanitary pad model has 1.1–2.3 times less potential environmental impacts than the conventional model). The most remarkable difference in environmental impact between the two models occurs in abiotic depletion of fossil fuels, photochemical oxidation, and water scarcity where the environmental impact of the conventional sanitary pad model is 2.3, 2.2, and 2.1 times higher than the impact of the Hempur sanitary pad, respectively. A detailed overview of the data on the potential environmental impact of the two sanitary pad models can be found in the supplementary material: Table 3.

### Scenario analysis

**Fig. 10:**
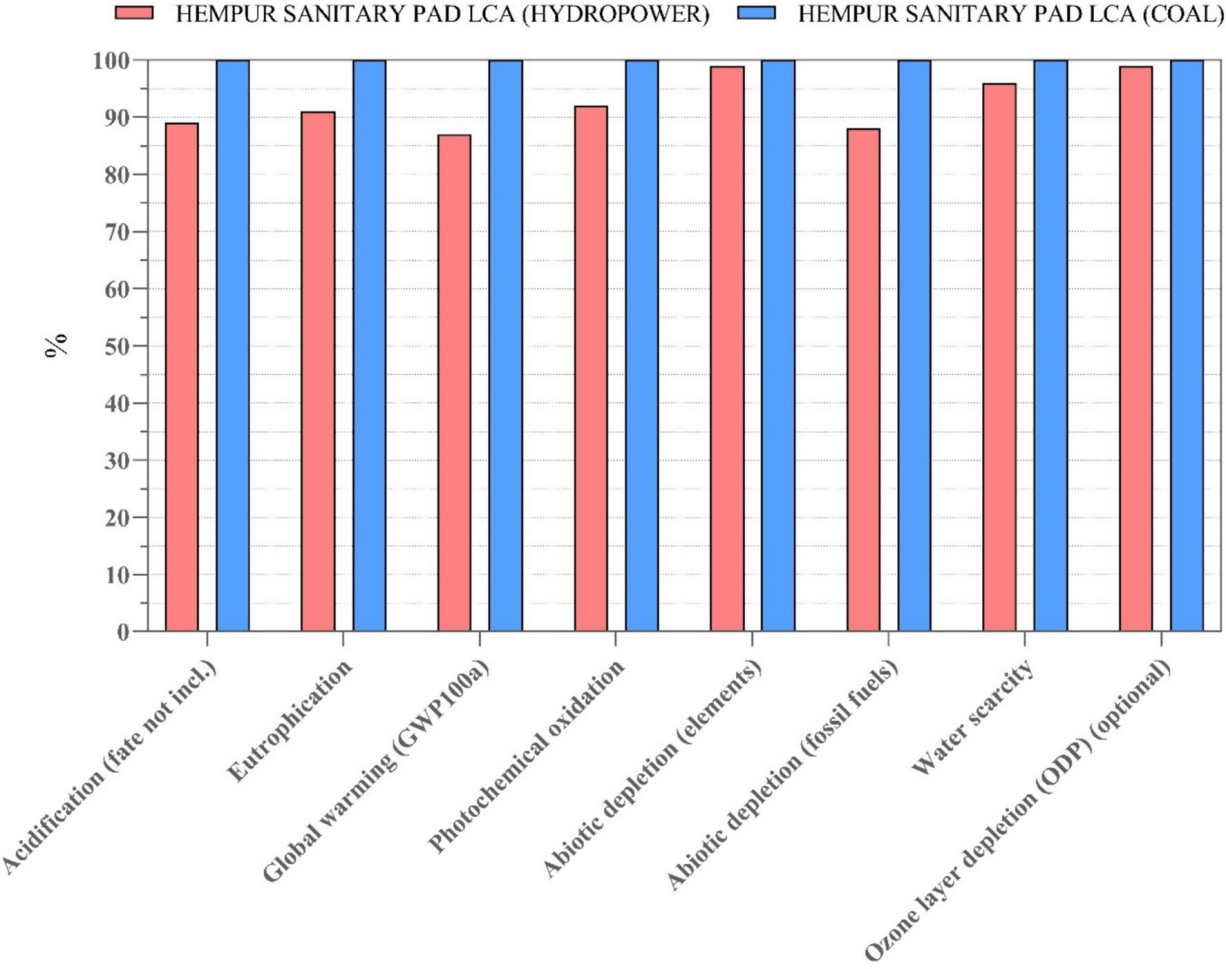
Characterized results of the whole life cycle of Hempur sanitary pad due to changes in electricity sources for bamboo pulp production

Scenario analysis for improvement is a valuable tool in LCA studies for exploring and evaluating potential strategies to reduce environmental impacts and promote sustainability across various sectors and industries. By considering alternative scenarios and assessing their implications, LCA practitioners can contribute to informed decision-making and the development of sustainable solutions. Based on Fisher International data, China’s pulp and paper industry heavily depends on coal as its main fuel source (Min 2020). In this study, it is also assumed that coal power is the source of electricity used for bamboo pulp production. Hence, a scenario analysis was conducted to analyze how environmental impacts during various stages of the life cycle would differ with the transition from the electricity provided by coal for pulp production to electricity from hydropower. The results of the comparison between the two energy sources for bamboo pulp production are shown in Fig. 10, highlighting changes in environmental impacts throughout the entire life cycle of Hempur sanitary pads.

The Hempur sanitary pad has shown a reduction of approximately 0.3–12% in all categories throughout its entire life cycle. Specifically, there is a 12.5% reduction in global warming. Moreover, there have been significant reductions in abiotic depletion of fossil fuels (11.4%) and acidification impacts (10.6%). Based on the previous results on the Hempur sanitary pad, the electricity from hard coal emits 2.2 kg CO_2_ eq in the upstream processes, whereas electricity from hydropower emits 1.7 kg CO_2_ eq, namely, 1.3 times more CO_2_ eq emissions produced by electricity which comes from hard coal than hydropower electricity. Since the production of bamboo pulp is connected to the upstream processes of the Hempur sanitary pad, the results indicate that when hydropower is utilized for pulp production, the upstream process of Hempur exhibits superior environmental performance across all impact categories, with reductions ranging from 1 to 30%. For instance, the upstream process with hydropower electricity caused a 30% reduction in acidification than upstream with electricity from coal. Moreover, in global warming, upstream processes by using hydropower electricity emit roughly 29% less than when pulp production uses electricity from hard coal. The supplementary material: Table 4 contains the characterized results of the upstream life cycle of the Hempur sanitary pad due to changes in electricity sources for bamboo pulp production.

### Cost barrier and market readiness

While bamboo-based sanitary pads demonstrate significant environmental benefits compared to conventional pads, their adoption may face challenges due to higher production costs. The cost of producing a conventional sanitary pad made from wood pulp includes expenses for materials such as wood pulp (0.0095 USD per pad), polyethylene sheet for the bottom layer (0.0041 USD per pad), and non-woven fabric for the top layer (0.0044 USD per pad). These costs total approximately 0.018 USD per pad for the core materials, excluding energy, transportation, and manufacturing overheads. In contrast, bamboo pulp, used as the primary raw material in bamboo-based sanitary pads, incurs higher costs due to its cultivation, transportation, and processing into suitable forms for sanitary pad production. Furthermore, polylactic plastic, commonly used as a bioplastic in eco-friendly pads, is significantly more expensive than polyethylene sheets. Preliminary estimates suggest that the cost of materials for bamboo-based sanitary pads is approximately 20–30% higher than conventional alternatives. This cost premium is attributed to the limited production scale, additional processing requirements, and higher raw material costs (Uddin et al. 2020). Despite the current cost barrier, there is potential for cost reductions through economies of scale and advancements in manufacturing technologies. As the production of bamboo pulp and bioplastics expands and more manufacturers adopt these sustainable materials, the overall cost of eco-friendly sanitary pads is likely to decrease. Additionally, governmental subsidies or tax incentives for sustainable products could further mitigate the cost barrier and enhance affordability (Fourcassier et al. 2022).

The market readiness of biodegradable sanitary pads, particularly those made with bamboo pulp and polylactic acid plastic, varies significantly across regions due to cultural, economic, and infrastructural factors. Increasing demand for sustainable products and growing environmental awareness create a favorable market in higher-income countries. However, in developing countries, affordability and limited access to composting or biodegradable waste management systems may hinder adoption. Key challenges include higher production costs driven by raw material expenses and limited supply chains (Mahajan et al. 2021). Despite these barriers, biodegradable pads appeal to environmentally conscious consumers, especially in regions where sustainability is a key purchasing criterion (Peberdy et al. 2019). Effective education and marketing campaigns highlighting environmental benefits, such as reduced carbon footprints and biodegradability, could enhance consumer acceptance (Prima Citta et al. 2024). Policy support, including subsidies for sustainable innovations and tax incentives for manufacturers, could play a pivotal role in reducing costs and improving accessibility. Additionally, advancements in production technologies and supply chain expansion may address scalability challenges. Coordinated efforts among stakeholders are essential to overcoming these barriers and promoting the widespread adoption of sustainable menstrual products (Jurga et al. 2020).

### Implications of the study

This research fills a notable gap in the menstrual literature by providing actionable insights for manufacturers, policymakers, and researchers. The study offers a framework for optimizing product design for manufacturers by focusing on sustainable material substitutions and renewable energy use in production. The results underline policymakers’ crucial role in promoting eco-friendly alternatives and creating waste management systems that accommodate compostable or biodegradable materials. Their actions can significantly impact the industry’s sustainability. Finally, for researchers, the study provides a robust methodology for evaluating the environmental impacts of other menstrual products. It paves the way for interdisciplinary assessments integrating social, economic, and health dimensions. This study advocates for a paradigm shift in the menstrual hygiene product industry toward more sustainable production practices by focusing on their environmental impacts. The adoption of bamboo-based materials could significantly reduce the environmental burden of menstrual products, thus aligning with global sustainability goals.

### Limitations and uncertainties

This study has some limitations. First, the data for the conventional pads were derived from a leading brand’s sustainability reports. While these are reliable sources, they may need to provide complete transparency or account for all production variables. Second, regional variations in energy mixes and waste management practices might influence the findings, limiting their generalizability to other contexts. Specifically, the geographic scope of this study is Sweden, where advanced energy systems and efficient waste management practices, such as incineration, are prevalent. This focus may only partially represent regions with less sustainable energy sources or waste management infrastructures. Third, while incineration was selected as the primary end-of-life treatment due to its dominance in Sweden’s waste management systems, alternative treatments such as composting or recycling, which may offer additional environmental benefits, were not included in the scope of this analysis. This limitation arises from Sweden’s need for more composting infrastructure for sanitary products. Future studies could explore these alternative scenarios to provide a more comprehensive assessment of the environmental performance of bamboo-based products. Fourth, the study assumes average transport distances and waste management scenarios. However, deviations in real-world practices, such as variations in transport distances or disposal methods, could result in different environmental outcomes.

Finally, no formal quantitative uncertainty analysis was conducted to evaluate how variations in key parameters (e.g., energy use, material sourcing) might affect the results. This was primarily due to constraints in the availability of detailed data for all life cycle stages and the scope of this study. However, the study’s robustness is supported by representative and validated data from sustainability reports and the ecoinvent database. Future research could include Monte Carlo simulations or sensitivity analyses to explore how assumption variations influence the findings, providing a more comprehensive understanding of their robustness.

## Conclusion

This study highlights the potential for improving the environmental performance of sanitary pads through the adoption of bamboo pulp as a key material. An attributional life cycle assessment was conducted to evaluate two types of sanitary pads, focusing on the life cycle stages and materials with the highest environmental impact. The results indicate that sanitary pads made from bamboo pulp generally have lower potential environmental impacts than conventional sanitary pads, particularly in categories such as photochemical oxidation, abiotic depletion of fossil fuels, and water scarcity. The findings suggest that the extraction and refinement of raw materials, alongside energy consumption during production, are the most impactful stages in the life cycle of both sanitary pad types. For conventional pads, materials like bleached wood pulp and low-density polyethylene were found to contribute over 80% of the overall impact in upstream processes. Conversely, while bamboo-based pads avoid these materials, the use of unbleached wood pulp and polylactic plastic still results in significant environmental impacts, particularly in terms of water scarcity and abiotic resource depletion. To enhance sustainability, the study recommends several concrete strategies for product design, sourcing, and manufacturing:

- Material substitution: Replace the unbleached wood pulp in bamboo-based pads with fully processed bamboo pulp or other sustainable alternatives, such as agricultural waste fibers (e.g., wheat straw or bagasse). This can reduce impacts associated with abiotic depletion and water scarcity.
- Optimized bioplastics: Explore more sustainable bioplastics for the back sheet, such as biodegradable polymers derived from algae or mycelium, which may offer lower environmental impacts compared to polylactic plastic.
- Localized sourcing: Shift toward localized bamboo sourcing and processing to reduce transportation-related emissions and costs. This approach can mitigate impacts in regions where bamboo cultivation is feasible.
- Circular economy initiatives: Integrate designs that facilitate composting or recycling, such as pads with fully biodegradable components, to improve end-of-life scenarios in regions lacking advanced incineration systems.
- Renewable energy adoption: Beyond transitioning bamboo pulp production to hydropower, manufacturers should aim for renewable energy adoption across all stages of production, including assembly and packaging.

Additionally, transitioning from coal to hydropower in the production of bamboo pulp could significantly reduce global warming and acidification impacts by approximately 30%. Despite these findings, several limitations were identified. For instance, the geographic scope of this study, focused on Sweden’s advanced waste management systems, limits generalizability to other contexts. Additionally, assumptions regarding transport distances and end-of-life treatments could introduce variability in the results. These limitations underline the importance of exploring alternative scenarios in future research.

While this study underscores the environmental benefits of bamboo pulp–based sanitary pads, it also highlights the need to address economic and social barriers to widespread adoption. Plant-based alternatives, including bamboo-based pads, are often associated with higher production costs due to raw material sourcing and manufacturing complexities. Furthermore, consumer acceptance and affordability remain critical factors, particularly in regions with lower purchasing power. Efforts to improve awareness about the environmental benefits of plant-based sanitary pads and to reduce production costs through technological innovations and economies of scale will be essential for broader market penetration. Finally, life cycle assessment provides a valuable framework for identifying environmental trade-offs but has limitations in addressing local impacts and non-quantifiable factors. Further research is recommended to build on these findings and explore innovative materials, scalable waste management strategies, and interdisciplinary assessments that integrate environmental, economic, and social dimensions for sustainable sanitary products.

## Supplementary Information

Below is the link to the electronic supplementary material.

Supplementary file1 (DOCX 24 KB)

## Data Availability

The data that support the findings of this study are available from the corresponding author upon reasonable request.
