# Supplementary material for: Toward eco-friendly menstrual products: a comparative life cycle assessment of sanitary pads made from bamboo pulp vs. a conventional one
Source: Environ Sci Pollut Res Int. 2025 Mar 18;32(14):9050–67. doi: 10.1007/s11356-025-36269-8 (PMC11968503; doi:10.1007/s11356-025-36269-8)
Supplement: Supplementary file 1 — Supplementary file1 (DOCX 24 KB) [file 11356_2025_36269_MOESM1_ESM.docx]

**Supplementary Material (SM)**

**Towards Eco-Friendly Menstrual Products: A Comparative Life Cycle Assessment of Sanitary Pads Made from Bamboo Pulp vs. a Conventional One**

1. **Table 1 contains the potential environmental impact of Hempur sanitary pads by life cycle stage:**

| Table 1: Potential environmental impact of Hempur sanitary pad divided per life cycle stages | | | | | |
| --- | --- | --- | --- | --- | --- |
| Impact category | Unit | Total | Upstream processes | Core processes | Downstream processes |
| Acidification (fate not incl.) | kg SO_2_ eq | 0.025 | 0.011 | 0.011 | 0.0005 |
| Eutrophication | kg PO_4_ eq | 0.006 | 0.003 | 0.001 | 0.0005 |
| Global warming (GWP100a) | kg CO_2_ eq | 3.981 | 2.221 | 0.776 | 0.983 |
| Photochemical oxidation | kg NMVOC | 0.021 | 0.008 | 0.012 | 0.0006 |
| Abiotic depletion, elements | kg Sb eq | 1.073E-05 | 9.14E-06 | 1.41708E-06 | 1.65118E-07 |
| Abiotic depletion, fossil fuels | MJ | 37.685 | 27.088 | 10.007 | 0.588 |
| Water scarcity | m^3^ eq | 1.920 | 1.726 | 0.033 | 0.159 |
| Ozone layer depletion | kg CFC-11 eq | 2.52E-07 | 1.25E-07 | 1.2004E-07 | 6.74947E-09 |

1. **Table 2 contains the potential environmental impact of conventional sanitary pads by life cycle stage:**

| Table 2: Potential environmental impact of conventional sanitary pad model divided per life cycle stages | | | | | |
| --- | --- | --- | --- | --- | --- |
| Impact category | Unit | Total | Upstream processes | Core processes | Downstream processes |
| Acidification (fate not incl.) | kg SO_2_ eq | 0.028 | 0.012 | 0.015 | 0.0003 |
| Eutrophication | kg PO_4_ eq | 0.010 | 0.004 | 0.005 | 0.0004 |
| Global warming (GWP100a) | kg CO_2_ eq | 5.931 | 2.615 | 2.601 | 0.714 |
| Photochemical oxidation | kg NMVOC | 0.047 | 0.012 | 0.033 | 0.0004 |
| Abiotic depletion, elements | kg Sb eq | 1.86074E-05 | 1.63685E-05 | 2.11904E-06 | 1.19805E-07 |
| Abiotic depletion, fossil fuels | MJ | 86.926 | 61.508 | 24.990 | 0.428 |
| Water scarcity | m^3^ eq | 4.118 | 3.748 | 0.277 | 0.093 |
| Ozone layer depletion | kg CFC-11 eq | 3.6388E-07 | 1.26442E-07 | 2.32538E-07 | 4.90023E-09 |

1. **Table 3 contains data on the potential environmental impact of the two sanitary pad models:**

| **Table 3:** Potential environmental impact of the two models of sanitary pad | | | |
| --- | --- | --- | --- |
| Impact category | Unit | Hempur sanitary pad | Conventional sanitary pad |
| Acidification (fate not incl.) | kg SO_2_ eq | 0.0246 | 0.028 |
| Eutrophication | kg PO_4_ eq | 0.006 | 0.011 |
| Global warming (GWP100a) | kg CO_2_ eq | 3.981 | 5.931 |
| Photochemical oxidation | kg NMVOC | 0.021 | 0.047 |
| Abiotic depletion, elements | kg Sb eq | 1.073E-05 | 1.860E-05 |
| Abiotic depletion, fossil fuels | MJ | 37.685 | 86.927 |
| Water scarcity | m^3^ eq | 1.920 | 4.118 |
| Ozone layer depletion (ODP) | kg CFC-11 eq | 2.518E-07 | 3.639E-07 |

1. **Table 4 contains the characterized results of the upstream life cycle of the Hempur sanitary pad due to changes in electricity sources for bamboo pulp production:**

| **Table 4:** The characterized results of the upstream life cycle of the Hempur sanitary pad due to changes in electricity sources for bamboo pulp production | | | |
| --- | --- | --- | --- |
| Impact category | Unit | upstream Process of Hempur sanitary pad (by using hydropower in bamboo pulp production) | Current upstream Process of Hempur sanitary pad |
| Acidification (fate not incl.) | kg SO_2_ eq | 0.008 | 0.011 |
| Eutrophication | kg PO_4_ eq | 0.003 | 0.003 |
| Global warming (GWP100a) | kg CO_2_ eq | 1.721 | 2.221 |
| Photochemical oxidation | kg NMVOC | 0.006 | 0.008 |
| Abiotic depletion, elements | kg Sb eq | 9.06133E-06 | 9.14441E-06 |
| Abiotic depletion, fossil fuels | MJ | 22.790 | 27.088 |
| Water scarcity | m^3^ eq | 1.662 | 1.726 |
| Ozone layer depletion (ODP) (optional) | kg CFC-11 eq | 1.24224E-07 | 1.25006E-07 |
